# Mining TCGA database for gene expression in ovarian serous cystadenocarcinoma microenvironment

**DOI:** 10.7717/peerj.11375

**Published:** 2021-05-04

**Authors:** Youzheng Xu, Yixin Xu, Chun Wang, Baoguo Xia, Qingling Mu, Shaohong Luan, Jun Fan

**Affiliations:** 1 Department of Gynecology, Qingdao Municipal Hospital, Qingdao, China; 2 Department of Neurology, Qingdao Municipal Hospital, Qingdao, China

**Keywords:** Ovarian serous cystadenocarcinoma, Differentially expressed genes, Overall survival, Tumor microenvironment, Immune and stromal scores

## Abstract

**Background:**

Ovarian cancer is one of the leading causes of female deaths worldwide. Ovarian serous cystadenocarcinoma occupies about 90% of it. Effective and accurate biomarkers for diagnosis, outcome prediction and personalized treatment are needed urgently

**Methods:**

Gene expression profile for OSC patients was obtained from the TCGA database. The ESTIMATE algorithm was used to calculate immune scores and stromal scores of expression data of ovarian serous cystadenocarcinoma samples. Survival results between high and low groups of immune and stromal score were compared and differentially expressed genes (DEGs) were screened out by limma package. The Gene Ontology (GO), the Kyoto Encyclopedia of Genes and Genomes (KEGG) pathway enrichment analysis and the protein-protein interaction (PPI) network analysis were performed with the g:Profiler database, the Cytoscape and Search Tool for the Retrieval of Interacting Genes (STRING-DB). Survival results between high and low immune and stromal score groups were compared. Kaplan-Meier plots based on TCGA follow up information were generated to evaluate patients’ overall survival.

**Results:**

Eighty-six upregulated DEGs and one downregulated DEG were identified. Three modules, which included 49 nodes were chosen as important networks. Seven DEGs (*VSIG4, TGFBI, DCN, F13A1, ALOX5AP, GPX3, SFRP4*) were considered to be correlated with poor overall survival.

**Conclusion:**

Seven DEGs (*VSIG4*, *TGFBI*, *DCN*, *F13A1*, *ALOX5AP*, *GPX3*, *SFRP4*) were correlated with poor overall survival in our study. This new set of genes can become strong predictor of survival, individually or combined. Further investigation of these genes is needed to validate the conclusion to provide novel understanding of tumor microenvironment with ovarian serous cystadenocarcinoma prognosis and treatment.

## Introduction

Ovarian cancer is one of the leading causes of female deaths worldwide. It is often diagnosed at late stage due to nonspecific symptoms (Allemani et al., 2015). Epithelial ovarian cancer (EOC) mainly includes serous, mucinous, endometrioid, clear cell, undifferentiated and other histological type of carcinoma. Ovarian serous cystadenocarcinoma (OSC) is the most deadly female cancer of reproductive system. It is estimated that 150,000 women die of this disease annually and 230,000 are diagnosed with ovarian cancer every year (Siegel, Miller & Jemal, 2016). Ovarian serous cystadenocarcinoma occupies about 90% of all ovarian cancers (Cancer Genome Atlas Research Network, 2011). 5-year survival of stage I patients is higher than 90%. In stages III to IV, less than 20%. Standard therapy includes cytoreductive surgery with first-line combination chemotherapy. Studies showed that 25% of ovarian cancer patients have primary resistance to chemotherapy regimens, 80% of patients may develop secondary resistance during chemotherapy (Zhang et al., 2019). With the development of technology, diagnostic biomarkers and targeted therapy has been applied in many kinds of cancers including EOC. Effective and accurate biomarkers for diagnosis, outcome prediction and personalized treatment are needed urgently.

The Cancer Genome Atlas (TCGA) have been established to discover genomic abnormalities in cancer of large cohorts worldwide to investigate the mechanism of tumorigenesis and development. Ovarian serous cystadenocarcinoma was divided into four subtypes: differentiated, immunoreactive, mesenchymal and proliferative in the TCGA database, according to their gene expression profiles. EOC patients show the highest prevalence of *BRCA* mutations among all pathological types of ovarian cancer which correlates with EOC progression and prognosis (Pan & Xie, 2017).

Tumor microenvironment play an important role in tumor genesis and progression, which contains immune cells, mesenchymal cells, endothelial cells, inflammatory mediators and extracellular matrix molecules (Hanahan & Weinberg, 2000). Stromal cells provide tumor cell growth signals, intermediate metabolites, and provide a suitable environment for tumor progression as well as metastasis (Yuan et al., 2016). An algorithm called ESTIMATE (Estimation of stromal and immune cells in malignant tumor tissues using expression data) calculates immune and stromal scores to predict the infiltration of non-tumor cells, by analyzing specific gene expression signature of immune and stromal cells, has been developed to analyze tumor purity and immune characteristics in TCGA database in several kinds of cancers (Alonso et al., 2017; Priedigkeit et al., 2017; Yoshihara et al., 2013).

In this study, we use ESTIMATE algorithm to identify key genes in OSC patients by analyzing TCGA expression profiles and clinical data. Further bioinformatic analysis were performed to determine the association of these genes with prognosis in ovarian cancer.

## Material and Methods

### Database

Gene expression profile for OSC patients was obtained from the TCGA data portal (https://tcga-data.nci.nih.gov/tcga/) (Level 3, 2017-09-08), using Affymetrix HT_HG-U133A platform, by Broad Institute of MIT and Harvard University cancer genomic characterization center (Tomczak, Czerwinska & Wiznerowicz, 2015). Clinical data of survival outcome (2016-04-27) and gene expression subtype (2016-05-27) were also downloaded from TCGA data portal. The ESTIMATE algorithm was used to calculate immune scores and stromal scores of expression data of ovarian serous cystadenocarcinoma samples (Yoshihara et al., 2013). Ordinary one-way ANOVA test was used to compare immune and stromal scores of different gene expression subtypes. Student *t* test was used to compare immune and stromal scores of BRCA mutation subtypes. Log rank test was used to compare survival results between high and low immune and stromal score groups.

### Data processing of DEGs

Limma package (http://bioconductor.riken.jp/packages/3.10/bioc/html/limma.html) was used to detect the DEGs between high and low groups of immune and stromal score. The cutoff criteria was set as adjusted *p* < 0.05 and |log2FC|>1.0. Each group owns unique DEGs. Venn diagram tool online (http://bioinfogp.cnb.csic.es/tools/venny) was used to analyze overlapping components.

### GO and KEGG pathway analysis of DEGs

We used standard GO analysis tools to divide gene functions into biological process (BP), molecular function (MF), and cellular component (CC) (The Gene Ontology , 2019). KEGG analyze genomes, biological pathways, diseases, chemical substances and drugs (Kanehisa et al., 2019). The g:Profiler database (https://biit.cs.ut.ee/gprofiler/) was used to identify the pathways and functional annotation of found genes and visualization of results. *p* < 0.05 was considered statistically significant (Reimand et al., 2019).

### PPI network and module analysis

Search Tool for the Retrieval of Interacting Genes (STRING-DB) database (http://string-db.org/) was used to evaluate the relationships among DEGs (Szklarczyk et al., 2019; Von Mering et al., 2005). The combined score is used as a cut-off value to limit the number of interactions of higher confidence. It is computed by correcting randomly observing probability before combining them from different evidence channels (Von Mering et al., 2005). The combined score was set as >0.4. PPI networks were constructed using the Cytoscape software (Doncheva et al., 2019). Nodes with higher degree of connectivity tend to be more essential and molecular complex detection module (MCODE) was then used to find clusters based on topology, which will help to locate key network and pathways in PPI network (Doncheva et al., 2019).

### Overall survival curve

By analyzing clinical data from TCGA ovarian serous cystadenocarcinoma database, Kaplan–Meier plots were generated to evaluate patients’ overall survival of expression levels of DEGs. *p* < 0.05 was considered statistically significant.

## Results

### Immune scores and stromal scores are significantly associated with OSC subtypes

Gene expression profiles and clinical information of 469 OSC samples were downloaded from the TCGA database, with initial pathologic diagnosis made between 1992 and 2013. 304 gene expression subtype data were also retrieved, which contains 69 (22.7%) cases of differentiated subtype, 84 (27.6%) cases of immunoreactive subtype, 69 (22.7%) cases of mesenchymal subtype, 82 (27.0%) cases of proliferative subtype. 6 samples of 469 were validated to contain BRCA1 or BRCA2 mutation.

**Figure 1:**
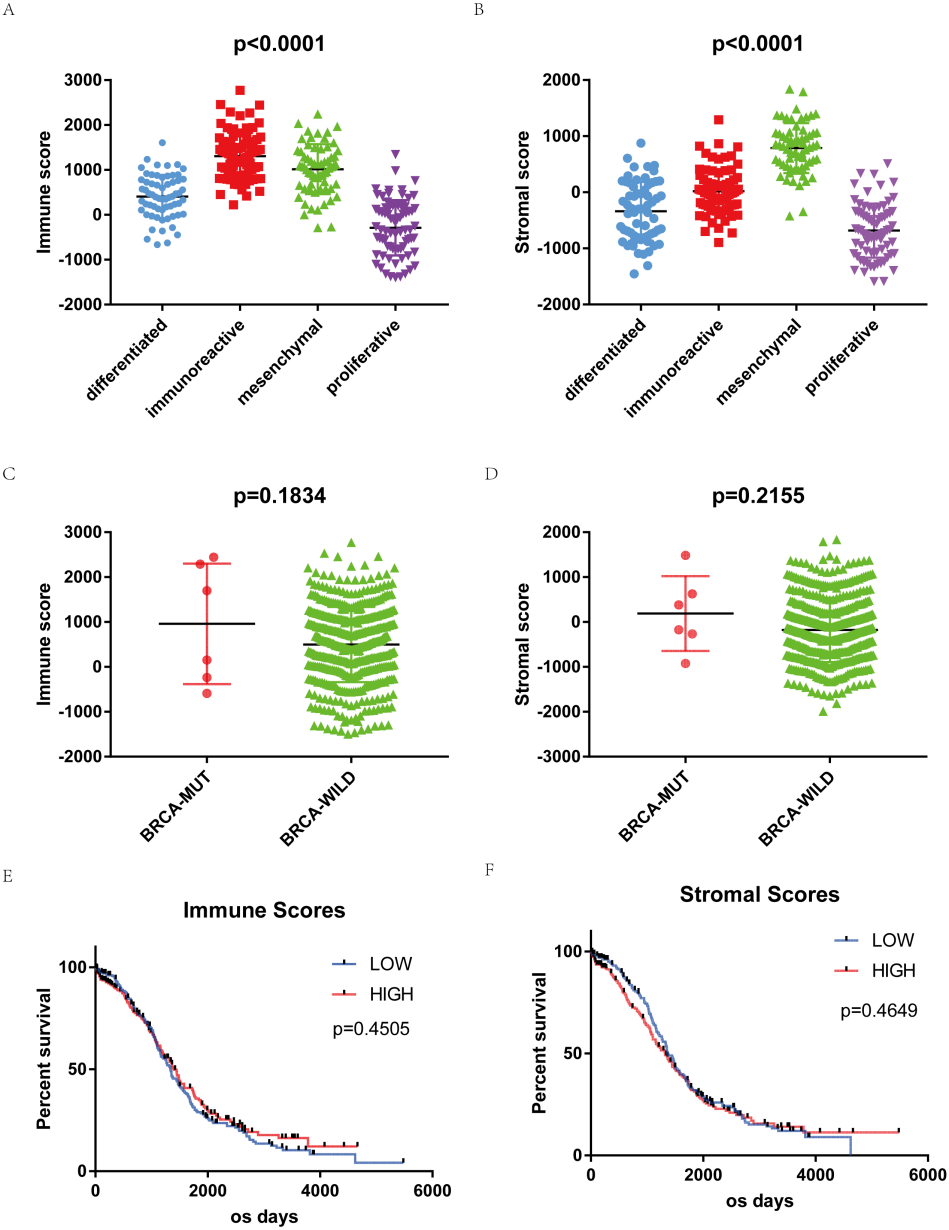
Immune scores and stromal scores are associated with OSC subtypes and their overall survival. (A) Distribution of immune scores of OSC subtypes. Box-plot shows that there is significant association between OSC subtypes and the level of immune scores (*n* = 304, *p* < 0.001). (B) Distribution of stromal scores of OSC subtypes. Box-plot shows that there is significant association between OSC subtypes and the level of stromal scores (*n* = 304, *p* < 0.001). (C) Distribution of immune scores for BRCA mutant and BRCA wildtype OSC cases. Box-plot shows that there is no significant association between BRCA mutation status and immune scores (*n* = 469, *p* = 0.1834). (D) Distribution of stromal scores for BRCA mutant and BRCA wildtype cases. Box-plot shows that there is no significant association between OSC subtypes and the level of stromal scores (*n* = 469, *p* = 0.2155). (E) Kaplan–Meier survival curves showed that median overall survival of cases with the high score group of immune scores is longer than the cases in the low score group (1399 d vs. 1336 d, *p* = 0.4505 in log-rank test). (F) Cases with lower stromal scores also showed longer median overall survival (1373 d vs. 1321 d, *p* = 0.4649 in log-rank test).

ESTIMATE algorithm was used to calculate immune and stromal scores of downloaded samples. Immune scores were distributed between −1498.58 to 2774.16, stromal scores −1988.05 to 1837.43, respectively. The average immune score of immunoreactive subtype cases ranked the highest of all 4 subtypes. The mesenchymal subtype has the highest stromal scores. The proliferative subtype cases had the lowest immune and stromal scores (Figs. 1A, 1B, *p* < 0.0001). Immune scores and stromal scores are correlated with the subtype classification of gene expression. The distribution of immune scores and stromal scores based on the status of BRCA1/2 mutation showed mutant samples have higher immune scores and stromal scores, although statistically not significant (Figs. 1C, 1D).

469 OSC samples were divided into high and low score groups (234:235) based on immune scores and stromal scores to compare their overall survival. Kaplan–Meier survival curves showed that median overall survival of cases of the high score group of immune scores is longer than the cases in the low score group (1399 d vs. 1336 d, *p* = 0.4505 in log-rank test). Cases with lower stromal scores also showed longer median overall survival (1373 d vs. 1321 d, *p* = 0.4649 in log-rank test), although both were not statistically significant (Figs. 1E, 1F).

### DEGs between groups of immune and stromal scores in OSC

**Figure 2:**
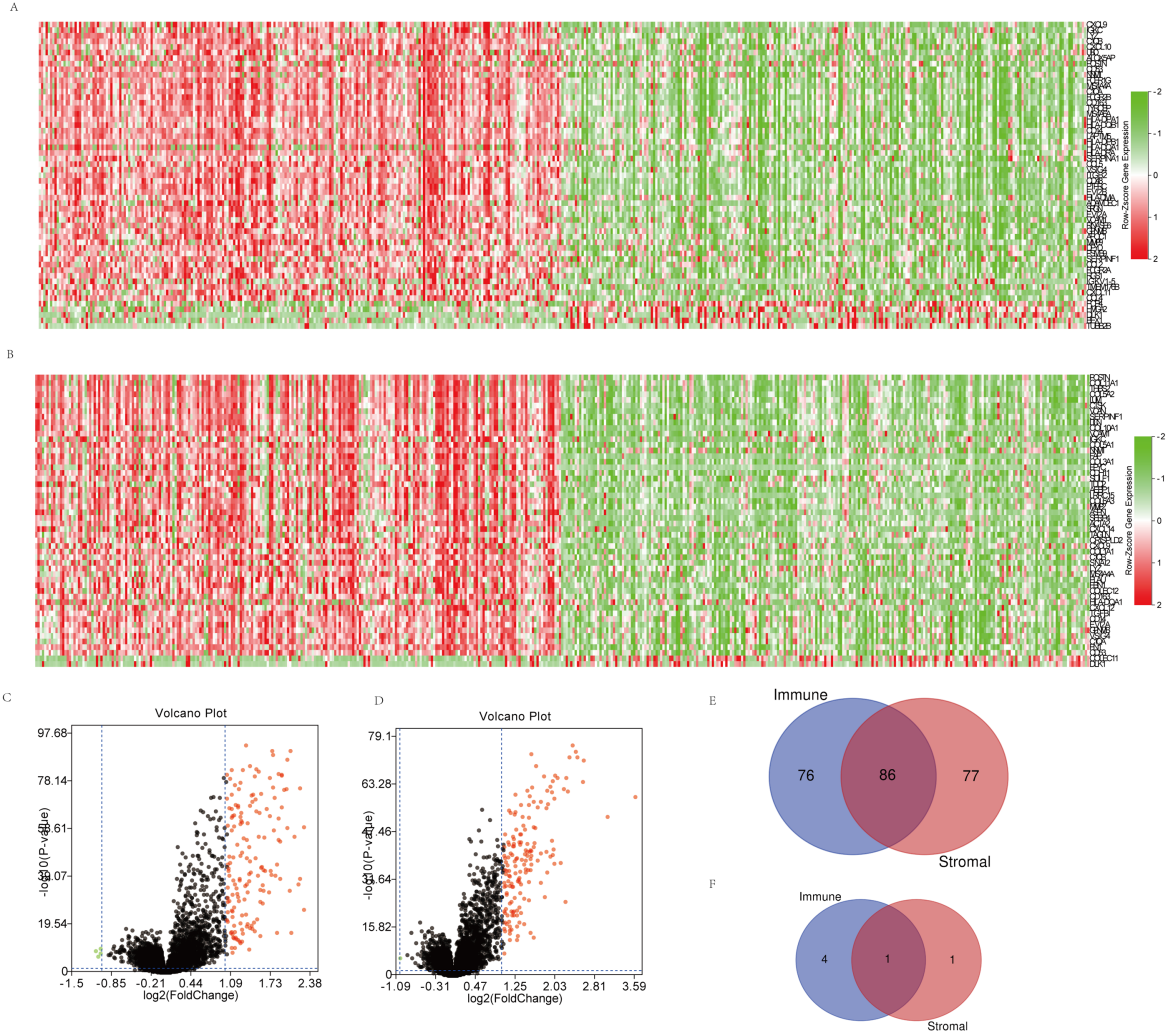
Comparison of gene expression profile with immune scores and stromal scores in OSC. (A, B) Distinct gene expression profiles between cases of high and low immune (A) or stromal (B) scores. (C, D) Volcano plots of distinct gene expression profiles between cases of high and low immune (C) or stromal (D) scores. (E, F) Venn diagrams showing the number of commonly upregulated (E) or downregulated (F) DEGs in immune and stromal score groups.

Microarray data of all 469 cases obtained in TCGA database were analyzed using limma package. Heatmaps and volcano plots in Fig. 2 showed distinct gene expression profiles between cases of high and low immune scores (Figs. 2A, 2C) and stromal scores (Figs. 2B, 2D) groups. 162 genes were upregulated and 5 genes downregulated in the high than the low immune score group, while 163 upregulated and 2 downregulated in the high than the low stromal score group (*p* < 0.05 and |log2FC|>1.0). Subsequently, Venn analysis was performed to get the intersection of the DEG profiles. Finally, 87 DEGs were significantly differentially expressed, of which 86 significantly upregulated (Fig. 2E) and 1 downregulated (Fig. 2F) in both immune and stromal comparison.

### GO function and KEGG pathway enrichment analysis of DEGs

**Figure 3:**
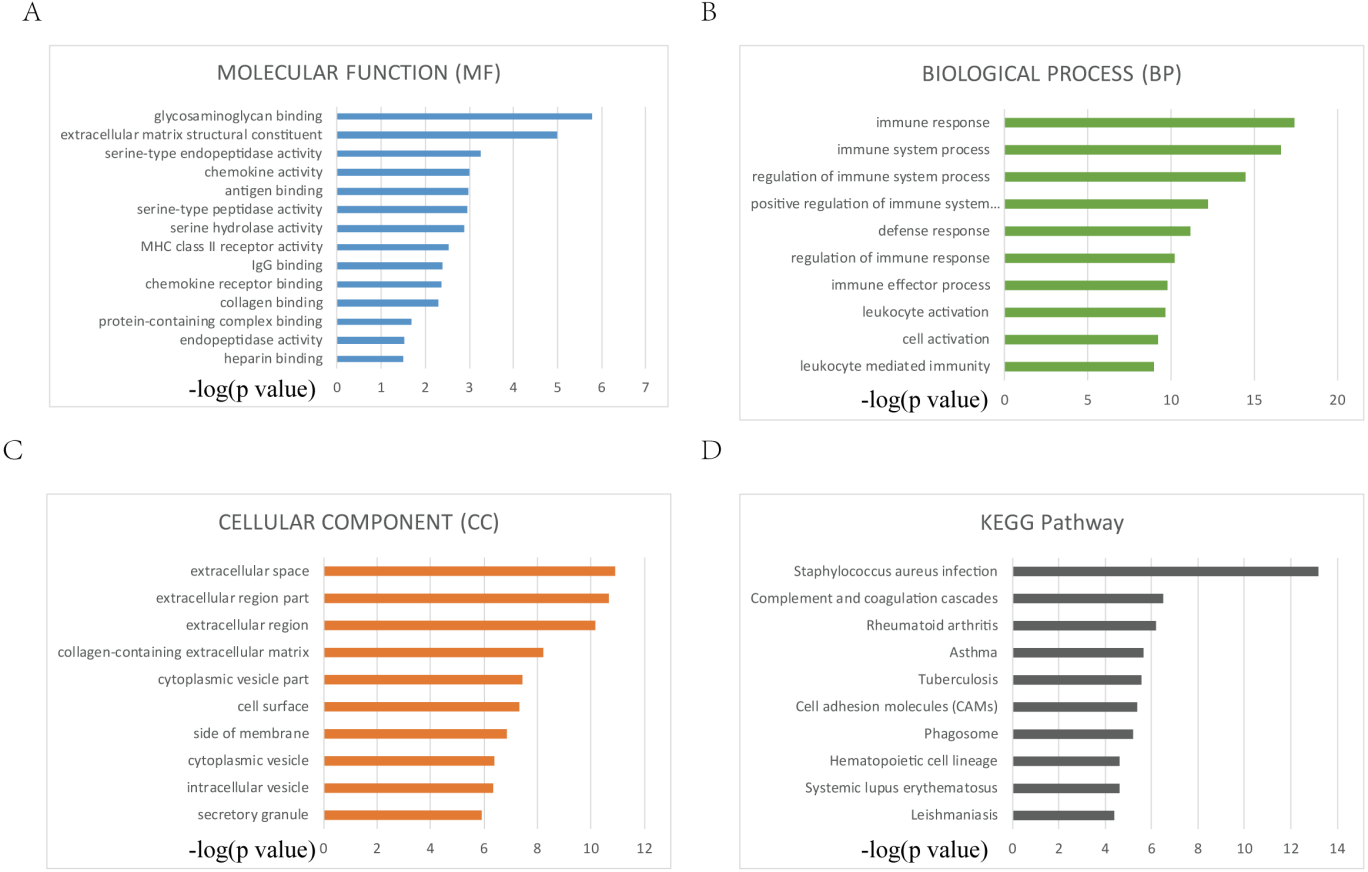
Top 10 GO and KEGG terms. (A, B, C, D) Top 10 GO and KEGG terms. False discovery rate (FDR) of GO analysis was acquired from g: Profiler annotation tool (*p* < 0.05).

The enriched GO terms were divided into MF, CC and BP ontologies. Top GO terms identified include glycosaminoglycan binding, immune response and extracellular space (Figs. 3A, 3B, 3C). DEGs were mainly enriched in staphylococcus aureus infection and complement and coagulation cascades according to the results of KEGG pathway analysis (Fig. 3D).

### PPI network analysis

**Figure 4:**
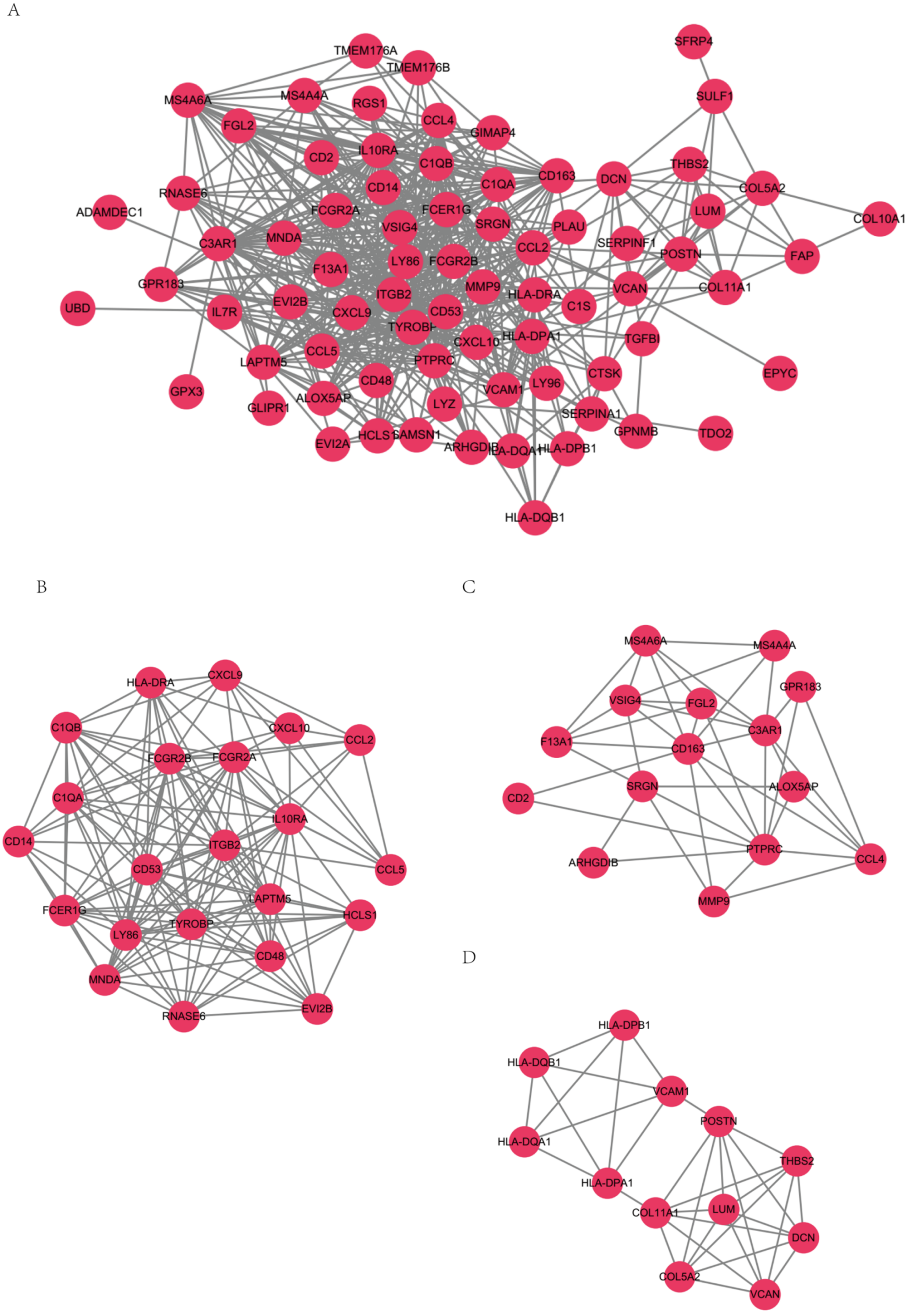
PPI networks. PPI networks (A) and 3 PPI networks of MCODE modules (B, C, D).

STRING-DB (score>0.4) results of PPI network were visualized by software Cytoscape, as presented in Fig. 4A. MCODE module was used to identify key genes of important pathways. The network was made up of 3 modules including 49 nodes (Figs. 4B, 3C, 4D). *TYROBP, ITGB2, C1QB, C1QA* and *CD53* in module B have the top connectivity among all DEGs and are considered to have important relationship to immune response in ovarian cancer. In module C, *CD163* had the most connections with other members of the module, which is reported to related to tumor associated macrophages and poor prognosis in ovarian cancer (Reinartz et al., 2014).

### Correlation of DEGs expression and overall survival

**Figure 5:**
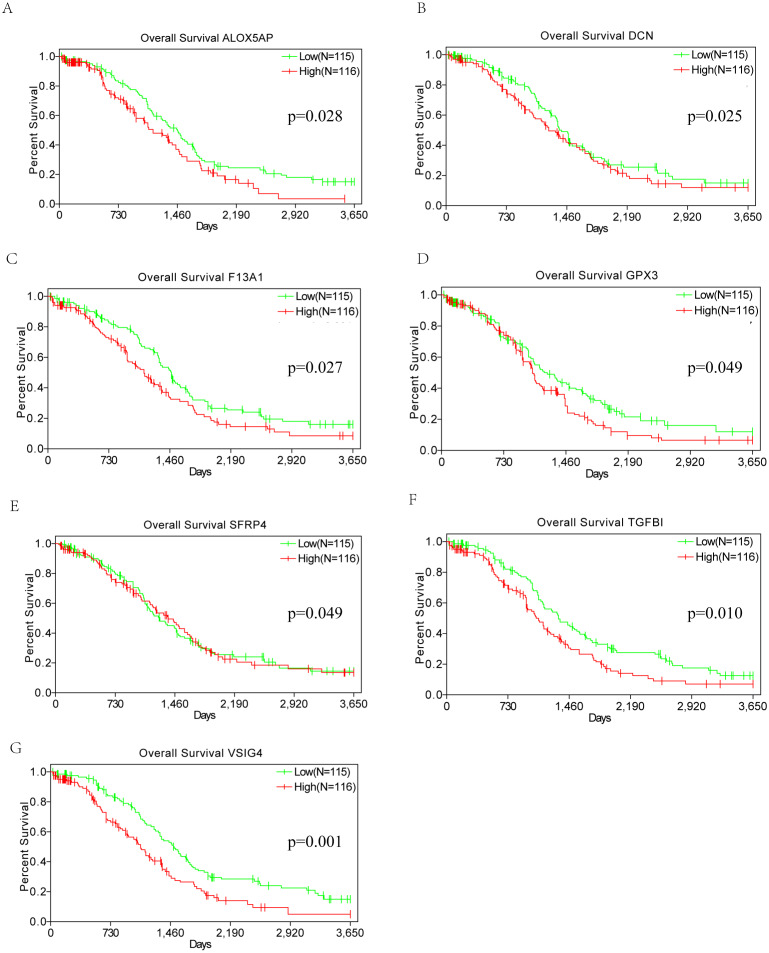
Kaplan–Meier survival curves. Kaplan–Meier survival curves of the correlation of expression of seven DEGs (A–G) with overall survival (*p* < 0.05, Log-rank test).

To evaluate the relationship of DEGs and overall survival of OSC patients from TCGA database, Kaplan–Meier survival analysis were performed based on the follow up information. Among the 86 DEGs upregulated in both the high-immune scores and high-stromal scores groups, 7 genes(*VSIG4, TGFBI, DCN, F13A1, ALOX5AP, GPX3, SFRP4*)considered to be correlated with poor overall survival in log-rank test (*p* < 0.05) (Fig. 5). The statistical results of Kaplan–Meier survival analysis were not convincing enough for the conclusion. Thus, the prognostic value of these 7 genes needs further investigation.

## Discussion

In the current work, we identified tumor microenvironment related genes that contribute to OSC overall survival in the TCGA database. Subsequently, by comparing gene expression between high and low immune and stromal scores, we extracted 86 upregulated genes involved in glycosaminoglycan binding, immune response and extracellular space, which is consistent with previous reports that the functions of immune cells and ECM molecules are related with tumor microenvironment (Yuan et al., 2016). By PPI network and MCODE module, TYROBP, *ITGB2, C1QB, C1QA, CD53* and *CD163* have top connectivity among all DEGs and are considered to have important relationship to immune response in ovarian cancer. High expression of 7 DEGs (*VSIG4, TGFBI, DCN, F13A1, ALOX5AP, GPX3, SFRP4*) were significantly correlated with poor overall survival in Kaplan–Meier survival analysis. However, the curve of *DCN*, *GPX3* and *SFRP4* contains some overlapping part between high and low expression groups (*p* value = 0.025, 0.049, 0.049 respectively). Also, the statistical results of Kaplan–Meier survival analysis were not convincing enough for the conclusion. Thus, the prognostic value of these 7 genes needs further investigation, which should be discussed and validated in experimental and clinical research in the future.

*VSIG4* (protein V-set and Ig domain-containing 4) is a novel B7 family-related macrophage protein. It can inhibit T-cell activation and promote cancer development. In advanced-stage and recurrent ovarian cancer patients, soluble VSIG4 levels were significantly increased (*p* = 0.0244 and 0.0288, respectively). High *VSIG4* expression in cancer tissue were associated with a longer disease-free interval (*p* = 0.0246) (Byun et al., 2017). Transforming growth factor-beta-inducible gene-h3 (*TGFBI*) is a secreted protein and possess tumor suppressor function. It activates integrin-mediated FAK and RhoA to sensitize ovarian cancer cells to paclitaxel in the extracellular matrix (Ahmed et al., 2007). *TGFBI* has been proved to be related with paclitaxel-resistance in ovarian cancer, which might be a potential therapeutic target for the enhancement of responses to chemotherapy in ovarian cancer patients (Wang et al., 2012). Another study suggested that *TGFBI* possesses both tumor suppressor and promoter function depending on the tumor microenvironment (Ween, Oehler & Ricciardelli, 2012). *DCN* (Decorin) is a member of the small leucine-rich proteoglycan family. It is important in collagen fibril assembly and tumor suppression. Studies in different cancer cell lines showed *DCN* effectively inhibits TGF-β induced cancer cell spreading and proliferation (Jarvinen & Prince, 2015). DCN was proved not only contributed to colonic carcinogenesis, but also is a novel potential biomarkers for the diagnosis of colon cancer (Li et al., 2017), however its function in ovarian cancer has not been investigated. F13A1 was proved significantly higher concentration in ovarian cancer plasma compared to normal female woman. Plasma proteins from ovarian cancer patients may be a powerful tool for clinical diagnosis and prognosis prediction which needs further investigation (Dufresne et al., 2018). *ALOX5AP* expression is increased in patients of obesity and insulin resistance (Elias et al., 2016) and recognized as a susceptibility gene for stroke (Papapostolou et al., 2014), but its role in cancer genesis and progression has not been investigated. High *GPX3* expression was associated with poorer overall patient survival and increased tumor stage. Exogenous oxidant insult as extracellular H_2_O_2_ can be removed via GPX3-dependent pathway in the microenvironment (Worley et al., 2018). *GPX3* shows an important protective and adaptation function in ovarian cancer cellular survival in the ascites tumor environment, especially in high grade serous adenocarcinoma (Worley et al., 2018). *SFRP4* inhibits Wnt signaling pathway and decreases transcription of Wnt target genes, Axin2, CyclinD1 and Myc, reducing migration ability of ovarian cancer cells by increasing the ability to adhere to collagen and fibronectin (Ford et al., 2013). Also *SFRP4* confers chemo-sensitization and improve chemotherapeutic efficacy (Deshmukh et al., 2017), which possesses both diagnostic and therapeutic potential in epithelial ovarian cancer.

## Conclusion

*VSIG4, TGFBI, DCN, F13A1, ALOX5AP, GPX3, SFRP4* were proved to be correlated with poor overall survival in our study. Further investigation of these genes is needed to validate the conclusion to provide novel understanding of tumor microenvironment with OSC prognosis and treatment.

##  Supplemental Information

10.7717/peerj.11375/supp-1 Supplemental Information 1 Immune and stromal scores of downloaded samples from the TCGA database

10.7717/peerj.11375/supp-2 Supplemental Information 2 GO AND KEGG Results of GO and KEGG pathway analysis of DEGs. The enriched GO terms were divided into MF, CC and BP ontologies. Top GO terms identified include glycosaminoglycan binding, immune response and extracellular space. DEGs were mainly enriched in staphylococcus aureus infection and complement and coagulation cascades according to the results of KEGG pathway analysis.

10.7717/peerj.11375/supp-3 Supplemental Information 3 DEGs DEGs screen out by limma package between the high and low immune score group with *p* value and logFC value.

10.7717/peerj.11375/supp-4 Supplemental Information 4 DEGs2 DEGs screen out by limma package between the high and low stromal score group with *p* value and logFC value.

## Additional Information and Declarations

### Data Availability

The following information was supplied regarding data availability: The raw measurements are available in the Supplementary Files.
